# Unraveling the Mystery of Taxol-Induced Cystoid Macular Oedema: Case Report and Literature Review

**DOI:** 10.22336/rjo.2025.02

**Published:** 2025

**Authors:** Radomir Babovic, Ben Burton, Nimesha Alex, Lakshmi Harihar, Tihomir Dugandzija

**Affiliations:** 1 Ophthalmology Department, James Paget Hospital, Great Yarmouth, University of East Anglia, Norwich, United Kingdom; 2 Oncology Department, Norfolk and Norwich University Hospital, Norwich, United Kingdom; 3 School of Medicine, University of Novi Sad, Oncology Institute of Vojvodina, Sremska Kamenica, Serbia

**Keywords:** Paclitaxel, cystoid macular oedema, breast cancer, side effects, CMO = cystoid macular oedema, TDICMO = taxane-drug induced cystoid macular oedema, ER = estrogen receptor, HER2 = human epidermal growth factor receptor 2, BCVA = best corrected visual acuity, VA = visual acuity, PH = pinhole, RE = right eye, LE = left eye, OCT = optical coherence tomography, RPE = retinal pigment epithelium, CAI = carbonic anhydrase inhibitors, VEGF = vascular endothelial growth factor, μm = micrometre

## Abstract

**Objectives:**

The primary aim of this article is to present cystoid macular oedema as one of the side effects of Paclitaxel (Taxol) chemotherapy. Paclitaxel is used as a treatment option in patients with different types of solid carcinomas. The potential loss of vision, already altered by the disease, further compromises their quality of life, a contributing factor to overall psychological and mental decline.

**Case presentation:**

A 69-year-old woman developed a drop in visual acuity that was painless, bilateral, and accompanied by wavy lines. This occurred six months after starting Paclitaxel chemotherapy for metastatic breast cancer. The diagnosis of cystoid macular oedema caused by Paclitaxel was made. The visual acuity significantly improved after Paclitaxel was discontinued, and the symptoms subsided.

**Discussion:**

Paclitaxel is a chemotherapy drug used to treat various types of cancers and has been associated with cystoid macular oedema (CMO) in rare cases. CMO is thought to result from the disruption of the normal blood-retinal barrier. The specific mechanism remains incompletely understood, and multiple mechanisms have been postulated. In typical CMO, leakage from parafoveal capillaries is demonstrated on fluorescein angiograms in a classic petaloid pattern. However, in Taxane-Drug Induced CMO (TDICMO), there is no evidence of fluorescein leakage on angiography. TDICMO is a rare drug side effect of breast cancer treatment, described just 14 times in the English literature.

**Conclusion:**

It is crucial to reiterate that if a patient undergoing Paclitaxel treatment experiences any vision changes, it is imperative to consult an ophthalmologist for a thorough evaluation and appropriate management. This step is essential for the patient’s well-being and to ensure the best possible outcome.

## Introduction

Paclitaxel, also known as Taxol, was isolated and produced from the plant Taxus brevifolia and was approved for medical use in the early 1990s. It is on the Essential Medicines List (EML), published by the World Health Organization (WHO), which contains the medications considered to be most effective and safest [1]. It is part of the taxane group of antineoplastic medicines used to treat ovarian, oesophageal, lung, breast, cervical, pancreatic, and Kaposi sarcoma. It is administered by intravenous injection and is used as monotherapy or alongside other chemotherapy [2]. Taxane agents, such as Paclitaxel, disrupt the microtubule network essential for mitosis within cells, ultimately resulting in cell death. Common side effects include hair loss, bone marrow suppression, numbness, allergic reactions, muscle pains, and diarrhoea [3].

Ophthalmological side effects secondary to Paclitaxel have been reported in 10% of the patients. These mainly include dry eye problems and optic neuropathy, but cystoid macular oedema (CMO) has also been described. However, only case reports of CMO can be found in the literature, and its incidence is unclear. Less than 30 cases of Taxane-Drug Induced CMO (TDICMO) were identified in the English literature. The onset of CMO can be from two months to several years after taxane treatment [4].

## Case report

At the beginning of 2021, a 69-year-old Caucasian woman with known metastatic breast cancer presented in the eye clinic with slowly progressive bilateral visual loss of 6 weeks’ duration. There was no history of previous eye problems or eye surgery. There was no other medical history except anxiety, for which the patient was using Propranolol tablets. At presentation, the patient had received 6 months of palliative chemotherapy with intravenous weekly Paclitaxel 80 mg/m2.

The patient was diagnosed with ER-positive HER2-negative metastatic breast cancer in 2017 and received multiple lines of palliative oncological treatment between 2017 and 2020, including endocrine therapy (Anastrozole, Exemestane), chemotherapy (Epirubicin, Capecitabine), bisphosphonate (Zoledronate), and breast/chest wall radiotherapy. Due to the slow but constant progression of the disease, in June 2020, she was started on weekly Paclitaxel 80 mg/m2. Six months later, she observed the gradual but constant deterioration of her vision, which led her to mention this to the oncology team, who referred her for an ophthalmology opinion.

On examination, the best corrected visual acuity (BCVA) was 6/20 Snellen in the right eye (RE) and 6/15 in the left eye (LE) and did not improve using pinhole (PH). The slit lamp examination showed clear cornea, white conjunctiva, no sign of inflammation in the anterior chamber, and the beginning of a cortical cataract, which could not explain the drop-in vision. Intraocular pressure was 17 mm Hg in both eyes on Goldmann applanation tonometry.

**Fig. 1:**
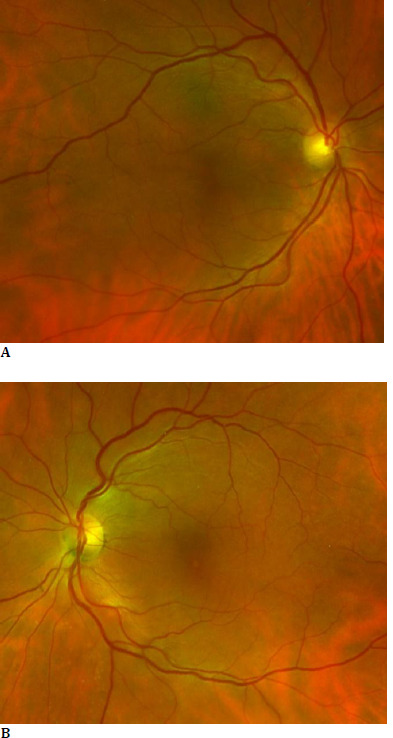
Fundus color photography of the right eye (**A**) and the left eye (**B**) using OPTOS retinal imaging: cystoid macular oedema causing bilateral loss of foveal reflex

**Fig. 2:**
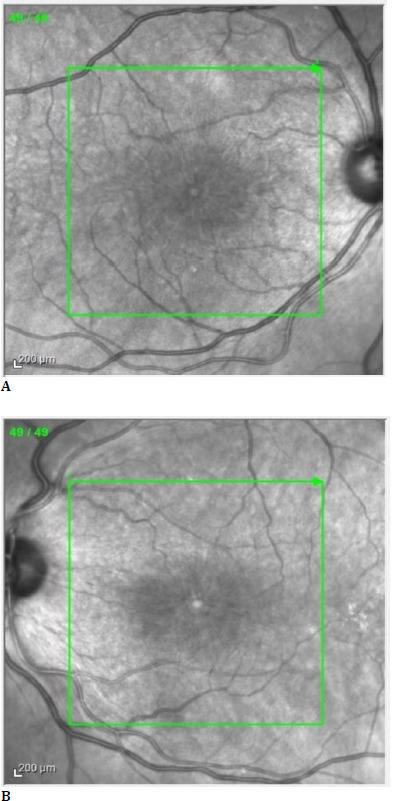
En-face OCT of the macula right (**A**) and left eye (**B**) with the petaloid appearance of the outer retina

**Fig. 3:**
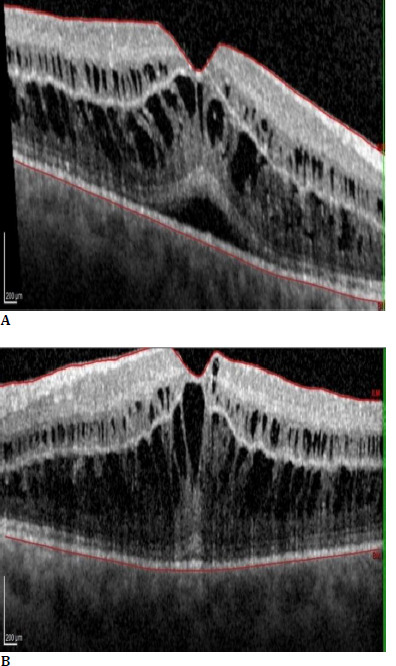
SD-OCT horizontal scan of the right eye (**A**) and left eye (**B**), showing intraretinal cysts

A dilated fundus ophthalmoscopy revealed bilateral loss of foveal reflex (**Fig. 1 A, B**). The en-face optical coherence tomography imaging (OCT Spectralis, Heidelberg Engineering, Germany) showed a petaloid arrangement of cystic cavities, which strongly suggested the presence of cystoid macular oedema (**Fig. 2 A, B**) [5]. Spectral-domain optical coherence tomography (SD-OCT) scans revealed an increase in macular thickness due to intraretinal accumulation of fluid with a central macular thickness (CMT) of 549 μm in the right eye and 612 μm in the left eye (**Fig. 3 A, B**).

**Fig. 4:**
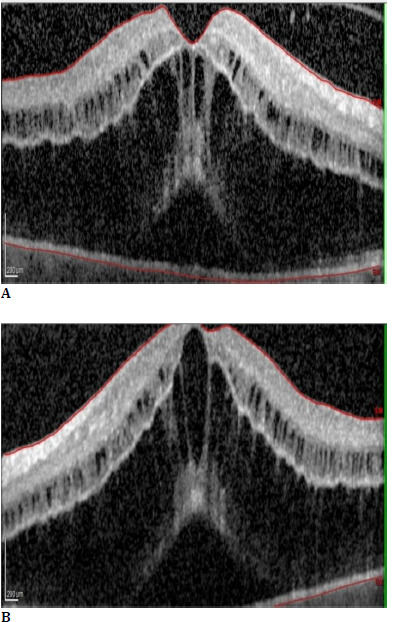
SD-OCT horizontal scan of the right eye (**A**) and left eye (**B**) showing an increase in the size of the retinal cysts during treatment with Ketorolac 0.5%

**Fig. 5:**
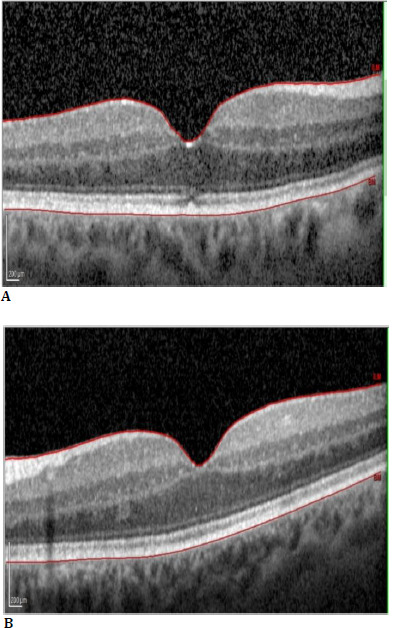
SD-OCT horizontal scan of the right eye (**A**) and left eye (**B**) shows complete resolution of the cystoid macular oedema after discontinuing paclitaxel treatment

As there were no signs of inflammation in the anterior or posterior segment of the eye, and no other medications were used except anxiety treatment, we concluded that Paclitaxel caused CMO. Due to the probable cause, the patient was diagnosed with TDICMO. Considering complex metastatic breast cancer, the patient was started on Ketorolac 0.5% drops 4xd in both eyes. Seven days later, despite treatment with Ketorolac drops, further worsening was evident. The best-corrected visual acuity has also dropped in the right eye (6/30) and the left eye (6/45). CMT has increased to 811 μm in RE and 911 μm LE, and VA dropped to 6/30 in both eyes (**Fig. 4 A, B**). After consulting with the oncology team and the patient, it was decided that the Paclitaxel treatment should be discontinued. Nine weeks after the initial ophthalmology diagnosis of TDICMO and eight weeks after discontinuation of the drug, CMO had entirely resolved. The CMT of the right macula was 303 μm and 310 μm in the left, with VA in RE 6/15 and VA in LE 6/15. After discontinuing Paclitaxel (**Fig. 5 A, B**), Eribulin chemotherapy was started in February 2021 with no complications recorded.

The initial diagnosis was confirmed when the CMO was resolved by stopping the Paclitaxel. Fluorescein angiography was considered and would have been performed for differential diagnosis if the CMO had not been determined.

The patient was reviewed on three occasions: on day 0, day 7, and day 67. The best corrected visual acuity (BCVA) and percentage improvement in VA closely mirrored the percentage change in central macular thickness (CMT) over time in both the right eye (**Table 1**) and the left eye (**Table 2**). The CMT exhibited a significant improvement after the discontinuation of Paclitaxel.

**Table 1:** Right eye best corrected VA and CMT at presentation and follow up; %, percentage; CMT, central macular thickness

Right eye	Visual acuity (Snellen)	Change (%) VA from day 0	CMT (μm)	Change (%) CMT from day 0
Day 0	6/20		549	
Day 7	6/30	- 33.3	811	+47.7
Day 67	6/15	+33.3	303	- 44.8

**Table 2:** Left eye best corrected VA and CMT at presentation and follow up; %, percentage; CMT, central macular thickness

Left eye	Visual acuity (Snellen)	Change (%) VA from day 0	CMT (μm)	Change (%) CMT from day 0
Day 0	6/15		612	
Day 7	6/45	- 66.6	911	+48.8
Day 67	6/15	0	310	- 49.3

## Discussion

Chemotherapy is an essential therapy for many cancers, including breast, lung, genitourinary, and head and neck cancers. Although much is known about adverse events related to taxanes, such as Docetaxel and Paclitaxel, information on the frequency of ocular adverse events, including their incidence, is scant. According to the British National Formulary (BNF), the side effects of the Paclitaxel drug have been divided into three groups. The group of common side effects is the biggest one. It includes numerous symptoms such as headache, hyperpyrexia, hypertension, increased risk of infection, influenza-like illness, insomnia, laryngeal pain, lymphoedema, malaise, movement disorders, mucositis, muscle complaints, nail discoloration, nail disorders, and gastrointestinal problems. The group of uncommon side effects includes allergic rhinitis, breast pain, dry mouth, dysphagia, ear pain, embolism, eye discomfort, facial swelling, hyperglycaemia, hypoglycaemia, hypotension, photosensitivity reaction, polydipsia, abnormal reflexes, swelling, syncope, and tinnitus. The last group is the group of rare side effects, including atrioventricular block, cardiac arrest, congestive heart failure, and left ventricular dysfunction [6]. Interestingly, CMO is not mentioned in any of these classifications.

Epiphora is the most reported ophthalmologic adverse reaction by women diagnosed with breast cancer and receiving treatment with taxanes. Epiphora is a self-limited, debilitating condition that disappears a few weeks after completing treatment or may be irreversible, and when present, it decreases the quality of life. Optic neuropathy can also decrease vision and quality of life, and diagnosis and treatment can affect patient outcomes [7]. Yamane et al. reported TDICMO as one of the rare drug side effects of breast cancer treatment, described just 14 times in the English literature [8]. In their literature review, Ye et al. found 24 cases of cystoid macular oedema caused by Paclitaxel [9]. All these adverse events have been reported as case reports, which cannot quantify the incidence or provide the magnitude of these events. We identified 50 cases by searching through PubMed and using the keywords “cystoid macular oedema”, “paclitaxel”, and “case report”, which is similar to the 57 cases that were identified by Alvarez et al. [10].

In 2020, over 2 million women across the globe were diagnosed with breast cancer. Chemotherapy involving Paclitaxel or Docetaxel is widely used as part of curative treatment for high-risk non-metastatic breast cancer and as part of palliative treatment for metastatic breast cancer. Ophthalmologists and oncologists must be well-versed in the frequency and nature of adverse ocular events due to taxane chemotherapy [11].

Taxanes are a type of chemotherapy drug that attaches to microtubules present in the cytoskeleton of cells. This attachment restricts microtubule remodeling and inhibits mitosis in rapidly dividing cancer cells. However, taxanes also interfere with the regular interaction between the extracellular matrix and cellular cytoskeleton. This interaction is crucial for regulating tissue interstitial pressure, which affects fluid movement out of capillaries. Due to this, peripheral oedema, a condition characterized by swelling in the extremities, is a common side effect of taxanes and can sometimes be severe [12,13].

TDICMO may present with a normal fluorescein angiogram, indicating little or no capillary leakage. This type of non-leaking CMO can also be observed in niacin-related maculopathy, certain forms of retinitis pigmentosa, juvenile X-linked retinoschisis, and Goldmann-Favre syndrome. Nonetheless, the exact cause of CMO without capillary leakage is yet to be fully understood. If anti-inflammatory treatments do not significantly improve, inflammatory causes may be excluded. The lack of evidence of fluorescein leakage and inadequate response to anti-VEGF intravitreal injection may point toward intact blood-retinal barriers [4].

According to Kuznetsova et al., when examining the OCT, the separation plane of TDICMO is found above the external limiting membrane. In TDICMO, the four external bands, clearly identified in inflammation-induced CMO, appear attenuated and not disrupted. This suggests that the fluid in TDICMO has a high viscosity, creating a shadow underneath. TDICMO likely originates from dysfunction in the retinal pigment epithelium and its impact on microtubule functions. The author describes TDICMO as a mystery due to the lack of evidence regarding its pathophysiology [14].

Other researchers suggest that taxanes’ direct toxicity on Muller cells may lead to ion regulation deterioration, which can cause cellular swelling and cystic formation. Furthermore, dysfunctional retinal pigment epithelium (RPE) due to taxane toxicity may also be a possible cause, as malabsorption across the RPE instead of vascular leakage could occur. Other studies propose that the fluid leaking rate might be too slow to be detected on fluorescein angiography or that only molecules smaller than fluorescein could selectively leak from capillaries [4,15,16].

Evidence shows that xanthophylls function as natural microtubule stabilizers in the primate retina by binding to the same site as Paclitaxel. However, it is unclear whether altering the carotenoid pathway can affect the development of TDICMO. Despite systemic Bevacizumab combined with taxane chemotherapy, there have been reports of TDICMO developing independently of the vascular endothelial growth factor pathway [17].

Treatments used for TDICMO include systemic or topical carbonic anhydrase inhibitors (CAI), topical anti-inflammatory agents, intravitreal Bevacizumab, and cessation of taxane therapy. The effectiveness of using topical Dorzolamide for taxane-related cystoid macular oedema is still uncertain. Carbonic anhydrase inhibitors may work by inhibiting the carbonic anhydrase enzyme at the basolateral membrane of the retinal pigment epithelium. This action could lead to a decrease in subretinal pH and an improvement in both subretinal and intraretinal fluid resorption by the RPE, potentially resulting in improved CMO [18]. Intravitreal anti-VEGF and intravitreal Dexamethasone have shown some but limited effects on TDICMO, according to Burgas et al. and Ye et al. [9,19]. Hassouna et al. suggested systemic acetazolamide combined with non-steroid inflammatory drops effectively treat TDICMO [12,20]. In the case we presented, the vision and CMO with non-steroid anti-inflammatory drops significantly worsened. The best treatment so far has been stopping the taxane drug when it is safe to do so.

## Conclusion

Taxane-induced cystoid macular oedema (TDICMO) is a recognized but rare complication of chemotherapy. Since its initial reports with Docetaxel in 2003 and Paclitaxel in 2007, approximately 50 cases of TDICMO have been documented in the literature. However, the underlying mechanisms of this condition remain poorly understood, and current theories fail to explain its development adequately. This case study highlights that macular oedema, a potential side effect of Paclitaxel therapy, can resolve spontaneously upon discontinuation of the drug. Despite the rarity of taxane-associated maculopathy, oncologists should remain alert to its occurrence. Patients receiving Paclitaxel should undergo ophthalmological evaluations if visual symptoms arise. The pathophysiology of TDICMO continues to be an unresolved area of investigation.
